# Identification and Characterization of *Mycoplasma feriruminatoris* sp. nov. Strains Isolated from Alpine Ibex: A 4th Species in the *Mycoplasma mycoides* Cluster Hosted by Non-domesticated Ruminants?

**DOI:** 10.3389/fmicb.2017.00939

**Published:** 2017-05-30

**Authors:** Chloé Ambroset, Corinne Pau-Roblot, Yvette Game, Patrice Gaurivaud, Florence Tardy

**Affiliations:** ^1^Université de Lyon, VetAgro Sup, UMR Mycoplasmoses des RuminantsMarcy-l'Étoile, France; ^2^Anses, Laboratoire de Lyon, UMR Mycoplasmoses des RuminantsLyon, France; ^3^Unité de Biologie des Plantes et Innovation, EA 3900, Université de Picardie Jules VerneAmiens, France; ^4^Laboratoire Départemental d'Analyses Vétérinaires de SavoieChambéry, France

**Keywords:** *Capra ibex*, *Mycoplasma feriruminatoris*, phylogenetic analysis, diversity, capsular polysaccharides, *M. mycoides* cluster

## Abstract

The genus *Mycoplasma*, a group of free-living, wall-less prokaryotes includes more than 100 species of which dozens are primary pathogens of humans and domesticated animals. *Mycoplasma* species isolated from wildlife are rarely investigated but could provide a fuller picture of the evolutionary history and diversity of this genus. In 2013 several isolates from wild Caprinae were tentatively assigned to a new species, *Mycoplasma* (*M.) feriruminatoris* sp. nov., characterized by an unusually rapid growth *in vitro* and close genetic proximity to ruminant pathogenic species. We suspected that atypical isolates recently collected from Alpine ibex in France belonged to this new species. The present study was undertaken to verify this hypothesis and to further characterize the French ibex isolates. Phylogenetic analyses were performed to identify the isolates and position them in trees containing several other mycoplasma species pathogenic to domesticated ruminants. Population diversity was characterized by genomic macrorestriction and by examining the capacity of different strains to produce capsular polysaccharides, a feature now known to vary amongst mycoplasma species pathogenic to ruminants. This is the first report of *M. feriruminatoris* isolation from Alpine ibex in France. Phylogenetic analyses further suggested that *M. feriruminatoris* might constitute a 4th species in a genetic cluster that so far contains only important ruminant pathogens, the so-called *Mycoplasma mycoides* cluster. A PCR assay for specific identification is proposed. These French isolates were not clonal, despite being collected in a restricted region of the Alps, which signifies a considerable diversity of the new species. Strains were able to concomitantly produce two types of capsular polysaccharides, β-(1→6)-galactan and β-(1→6)-glucan, with variation in their respective ratio, a feature never before described in mycoplasmas.

## Introduction

The genus *Mycoplasma* contains a group of free-living, wall-less prokaryotes characterized by small genomes (0.58–1.38 Mbp) with a low G + C content (23–40 mol%), small cell size and a strict exogenous-sterol nutritional requirement for growth. This genus includes more than 100 species of which the best studied are primary pathogens of humans (~6 species) and domesticated animals (~31 species) (Brown, 2010). In contrast the importance of mycoplasmas isolated from wildlife is often underestimated but could prove very useful to understand the overall evolutionary history of mycoplasmas, their capacity to adapt to different hosts and their real genetic diversity (Brown et al., 2005).

Recently several isolates collected from wild Caprinae, namely 4 Alpine ibex (*Capra ibex*) bred in Berlin zoo as well as one Rocky Mountain goat (*Oreamnos americanus*) in the USA, were tentatively assigned to a new mycoplasma species characterized by unusually rapid growth *in vitro* (Jores et al., 2013). The generation time of *Mycoplasma (M.) feriruminatoris* sp. nov. (27–29 min at 37°C) is an exceptional feature as the fastest species in the genus *Mycoplasma*, until then, had a generation time of about 3 h. Other phenotypical properties, as well as the genome sequencing of strain G5847^T^, further suggested that *M. feriruminatoris* sp. nov. was close to *M. mycoides* subsp. *capri*, one of the etiological agents of contagious agalactia (CA) in domestic goats (Manso-Silvan et al., 2007; Fischer et al., 2012). CA is a syndrome affecting small ruminants that causes important economic losses in the dairy industry and is listed as a notifiable disease by the World Organization for Animal Health (OIE). It includes typical clinical signs, such as mastitis, arthritis and keratoconjunctivitis as well as more exceptional ones like pneumonia and septicemia in young animals, or abortions (Corrales et al., 2007). Three taxa are actually responsible for CA in goats: *M*. *mycoides subsp. capri (Mmc), M. capricolum subsp. capricolum (Mcc)* and *M. putrefaciens*, while *M. agalactiae*, the historical etiological agent of CA, is found both in goats and sheep. *Mmc* and *Mcc* belong to the so-called *Mycoplasma* (*M*.) *mycoides* phylogenetic cluster that includes five closely related pathogenic (sub)species of ruminants (Cottew et al., 1987), the taxonomy of which was amended in 2009 (Manso-Silvan et al., 2009). This cluster also includes *M. mycoides subsp. mycoides* (*Mmm*) and *M. capricolum subsp. capripneumoniae* (*Mccp*), the causative agents of contagious bovine pleuropneumonia (CBPP) and contagious caprine pleuropneumia (CCPP), respectively, two other notifiable diseases on the OIE list. The 5th taxon of the cluster, *M. leachii*, is seldom isolated and is described as a bovine pathogen and a chimera between *mycoides* and *capricolum* species (Manso-Silvan et al., 2007; Tardy et al., 2009). Both its proximity to a cluster of taxa responsible for severe diseases and its rapid growth make *M. feriruminatoris* sp. nov. a very intriguing new species. Although it has not yet been reported from many countries, mycoplasma species from wild ruminants have been regularly described in different countries that have remained unidentified due to the time and cost required for a specific mycoplasma diagnosis (Nicolas et al., 2005; Gonzalez-Candela et al., 2007; Giangaspero et al., 2010). In the study by Gonzalez-Candela, unassigned mycoplasma species represented up to 6.2% of 321 sampled Spanish ibex (Gonzalez-Candela et al., 2007).

In France, between 2003 and 2011, several samples collected in the Savoy region from healthy live-captured animals and ibex carcasses were identified by membrane filtration dot-immunoblotting test (MF-dot) as *M. agalactiae* and *Mmc*, respectively, based on their antigenic profiles (Tardy et al., 2012). While the *M. agalactiae* ibex strains were characterized in detail and revealed the emergence of atypical strains, no further investigations were done with the strains classified as *Mmc* (Tardy et al., 2012). However, the work by Jores et al. (2013) made us aware that the rapid growth capacity of these isolates could indicate a possible misidentification of *M. feriruminatoris* as *Mmc*.

The present study was undertaken to check whether a sub-population of 27 of these ibex isolates originally identified as *Mmc* actually belonged to the *M. feriruminatoris* species. Both *M. feriruminatoris* G5847 type strain and 8756-C13 Rocky Mountain strain were included as controls (Manso-Silvan et al., 2007; Jores et al., 2013). Another strain, namely 283F08, isolated in Italy in 2005 from a severe keratoconjunctivitis case in an Alpine ibex, and identified as an atypical *Mmc* was also included in the study (Giangaspero et al., 2010). Comprehensive phylogenetic analyses were performed to accurately determine the taxonomic position of the ibex isolates with respect to the *M. mycoides* cluster and a specific PCR assay was developped. Lastly, the population diversity was genetically characterized by genomic macrorestriction and phenotypically by examining the capacity of different strains to produce capsular polysaccharides, a feature now known to distinguish species and serovars within the *M. mycoides* cluster (Bertin et al., 2015; Gaurivaud et al., 2016).

## Materials and methods

### *Mycoplasma* isolates, culture conditions, and identification

Twenty-seven mycoplasma isolates collected from Alpine ibex, and previously identified as *Mmc* by MF-dot (Tardy et al., 2012) were included in this study (Table 1). They were isolated within a restricted area of the French Alps delimited by the Italian frontier and the Gran Paradiso park (east) and by a line drawn between Modane, Champagny en Vanoise, and Peisey-Nancroix (west), between 2003 and 2011 (Tardy et al., 2012). All strains were grown for 24 h at 37°C in 5% CO_2_ in PPLO broth supplemented as previously described (Poumarat et al., 1991). When necessary, mycoplasmas were enumerated by plating serial dilutions of cultures onto solid medium, in triplicate, and counting the colonies after 24 h of incubation. *Mycoplasma* identification was performed by MF-dot (Poumarat et al., 1991) and by sequence analysis of a 781 bp locus of the *fusA* gene (Manso-Silvan et al., 2007; Maigre et al., 2008) that allows species identification using a phylogeny reconstruction tool provided on the leBIBI^QBPP^ website (https://umr5558-bibiserv.univ-lyon1.fr/lebibi/lebibi.cgi) (Flandrois et al., 2015). Strains G5847^T^ and 8756-C13 were added as reference *M. feriruminatoris* strains. The first one, isolated from the joint fluid of an Alpine ibex that had died in a zoo in Berlin in 1993, had been proposed as the type strain of the species and had its genome sequenced under GenBank accession no. ANFU00000000.1 (Fischer et al., 2013). The 8756-C13 strain was isolated by A.J. Da Massa in the USA before 1987 from a Rocky Mountain goat and originally identified as a *Mycoplasma spp*. by A. Breard in CIRAD (Manso-Silvan et al., 2007). Another unidentified mycoplasma strain (283F08) isolated from a *Capra ibex* suffering from keratoconjunctivitis in northern Italy (Valle d'Aosta) was kindly provided by Prof. M. Giangaspero and Dr. R. Ayling (Giangaspero et al., 2010).

**Table 1:** *****M. feriruminatoris*** sp. nov. isolates from French Alpine ibex included in this study**.

**Isolate no^a^**	**Isolation**			
	**Year**	**Source**	**Clinical context^b^**	**Co-isolation with other *Mycoplasma***
L13461	2003	Unknown	Septicemia	No
L14815	2007	Ear canal	K	*M. agalactiae* (lung)
L14915^*^		Eyes	K	*M. auris* (eyes)
L14924		Ear canal	P	No
L14822		Ear canal	P	No
L14798		Ear canal	Capture only	No
L14940	2008	Ear canal	Capture only	*M. agalactiae* (ear canal)
L14945^*^		Lung	P	No
L14976		Ear canal	K, P	*M. agalactiae* (ear canal)
L14978^*^		Ear canal	P	*M. agalactiae, M. auris* (ear canal)
L15023		Ear canal	P	No
L15181^*^		Ear canal	K	*M. agalactiae* (lung)
L15199^*^	2009	Ear canal	P	*M. agalactiae, M. auris* (lung)
L15220		Ear canal	P	No
L15259		Nares	Capture only	No
L15260^*^		Ear canal	Enterotoxemia	*M. agalactiae* (lung)
L15311^*^		Ear canal	P	No
L15373^*^	2010	Eyes	P	*M. agalactiae* (eyes)
L15399^*^		Ear canal	Capture only	*M. agalactiae* (ear canal)
L15403^*^		Ear canal	Capture only	*M. agalactiae* (ear canal)
L15407^*^		Eyes	P	*M. agalactiae* (lung)
L15423		Nares	Capture only	*M. agalactiae* (lung)
L15543^*^	2011	Ear canal	Capture only	*M. agalactiae* (ear canal)
L15541^*^		Ear canal	Capture only	*M. auris* (ear canal)
L15564		Ear canal	Capture only	No
L15566		Ear canal	Not known	No
L15568		Ear canal	Capture only	No

a *The asterisks indicate cloned isolates*.

b *The animals were either captured for sampling and released afterwards or found dead and necropsied for determining the most probable cause of death (K, Keratoconjunctivitis; P, Pneumonia)*.

Several mycoplasma strains from non-targeted species were used to test the specificity of the *M. feriruminatoris* D500_0332 PCR assay. Ten strains were selected per (sub)species belonging or close to the *M. mycoides* cluster (*Mcc, Mmc, Mmm, M. leachii, M. putrefaciens, M. yeatsii)* against 5 strains of other species usually isolated from ruminants but remote from the cluster (*M. bovis, M. agalactiae, M. ovipneumoniae*, and *M. arginini*).

### DNA extractions and PCR amplifications

Genomic DNAs were extracted from 2 ml cultures using the phenol-chloroform method (Chen and Kuo, 1993) or a commercial kit from Qiagen. Several PCR assays were run according to the originally developed protocols. They targeted either the *fusA* gene (Manso-Silvan et al., 2007) or one of the seven housekeeping genes included in the MSLT scheme developed by Fischer et al., namely *adk, gmk, gyrB, pdhC, pgI, recA*, and *rpoB* (Fischer et al., 2012). Due to amplification failure and following a personal recommendation by Jorg Jores, the *recA* primers were slightly modified to recAFnew 5′-ATGTTGAAACATTTTCATCAGG-3′ and RecARnew 5′-CTAATTCACCAAGTTTATCAATTCC-3′. Several new primers were designed using Primer3 in Geneious® software 8.0.5 (Untergasser et al., 2012) to specifically amplify (1) *Gsm* (“Glycan synthase of Mollicutes”) genes identified in *M. feriruminatoris* or previously described in *M. mycoides subsp. capri* PG3^T^ and *M. agalactiae* (Gaurivaud et al., 2016) and (2) the D500_0332 gene used for species identification of *M. feriruminatoris*. All newly designed primers are listed in Table 2 with the PCR assay conditions. When required, PCR products were sequenced using an external facility at Beckman Coulter Genomics (Genewiz, United Kingdom).

**Table 2:** **Primers used for PCR assays**.

**Primer name**	**Primer sequence 5′–3′**	**Target species**	**Target sequence**	**Size (pb)**	**PCR assay conditions**					
Ctg3_GT2_F	GCTACCAATATAACCAGCTC	*M. feriruminatoris*	*Gsm*	2218	94°C	95°	44°C	72°C	35 cycles	72°C
Ctg9_GT2_R	ATCCAGCACAAGGAATAATG				2 min	30 s	30 s	1 min 30 s		10 min
Ctg6_GT2_F	GTGAGGTATTTAAAAGAGTTGG	*M. feriruminatoris*	*Gsm*	1791	94°C	95°	51°C	72°C	35 cycles	72°C
Ctg20_GT2_R	GCAAGTGCTTAATAATAAGGTTA				2 min	30 s	30 s	1 min 30 s		10 min
GsmG5847_1&2F	TTATTCCAGCACATAATGAA	*M. feriruminatoris*	*Gsm*		94°C	95°	48°C	72°C	35 cycles	72°C
GsmG5847_Ctg6-20R	GTATTTGGTTTTGTTCACTT			1176	2 min	30 s	30 s	1 min		10 min
GsmG5847_Ctg3-9R	TATTTTGTACTTGAGCAACT			900						5 min
GsmPG3_0120F	TTAGAAGAACAAACCGTATT	*M. mycoides* subsp. *capri* PG3^T^	*Gsm MMC_0120*	930	94°C	95°	48°C	72°C	35 cycles	72°C
GsmPG3_0120R	GAATTAGCGAATGCAATATT				2 min	30 s	30 s	1 min		5 min
GsmPG3_6610F	AATCGTTACTGTATAAGTGG	*M. mycoides* subsp. *capri* PG3^T^	*Gsm MMC_6610*	1480	94°C	95°	48°C	72°C	35 cycles	72°C
GsmPG3_6610R	CAAGGAAGTGAGACTATAAG				2 min	30 s	30 s	1 min 30 s		10 min
Gsm14268_1260F	CCATGGTATGATAATAAGCA	*M. agalactiae*	*GsmA MAGb_1260*	739	94°C	95°	48°C	72°C	35 cycles	72°C
Gsm14628_1260R	AACAATAGGGAAGAGTAAAC				2 min	30 s	30 s	1 min		5 min
D500_0332F	GAAAGTATAATAACCCAGCT	*M. feriruminatoris*	D500_0332	765	94°C	94°	48°C	72°C	35 cycles	72°C
D500_0332R	TAGCTTGACTAGGATAATCA				2 min	30 s	30 s	1 min		5 min
**SUPPLEMENTARY INTERNAL PRIMERS USED FOR SEQUENCING ANALYSIS**										
Ctg3_GT2_F2	AAAGGTGGAAACTTTGCTAT	*M. feriruminatoris*	*Gsm*							
Ctg6_GT2_F2	AGTTGCAGATAATTGTACAGA									
Ctg9_GT2_R2	CCAACACTTCATCTCATTCT									
Ctg20_GT2_R2	TCCAACACATCATCTCATTC									

### Phylogenetic analysis

Sequence editing, alignment and concatenate constructions were performed using Seaview software (http://doua.prabi.fr/software/seaview) (Gouy et al., 2010) or Geneious® software. All sequences were aligned and trimmed to the exact amino acid length as used in the two MLST schemes developed for the *M. mycoides* cluster (Manso-Silvan et al., 2007; Fischer et al., 2012). For our phylogenetic analysis we applied the Bayesian approach implemented by MrBayes v3.2.6 as described in Fischer et al. (2012) with a few modifications and used the IQ-Tree web server (http://iqtree.cibiv.univie.ac.at/), instead of the jmodeltest program, to select the best fitting model of nucleotide or amino acid substitution. In the end the selected model was identical, *i.e*. the Generalize Time Reversion (GTR) model and Gamma-distributed rates across sites (G) with an additional invariable sites (I) parameter for both MLST and *fusA*. The Codon option and the standard 4 by 4 nucleotide substitution model were chosen with a maximum of 15 million and 35 million iterations for the *fusA* and the MLST scheme analyses, respectively (instead of 10 millions) to reach chain convergences. The first 40% (instead of 10%) of each chain was removed for burn-in. Chain convergence was estimated when posterior probabilities <0.005 were reached (instead of using a specific program). The majority consensus trees were drawn using Figtree v1.4.2 (http://tree.bio.ed.ac.uk/software/figtree/). The genome from *Mesoplasma florum* (NC_006055.1) was used as an outgroup and nucleotide sequences (both *fusA* and the 7 genes of the MLST scheme) from strains 8756-C13 (Fischer et al., 2012), *M. mycoides subsp. mycoides* Gladysdale and PG1^T^ (CP002107, NC_005364), *M. mycoides subsp. capri* PG3^T^ and GM12 (NZ_JFAE00000000.1, CP012387), *M. capricolum subsp. capricolum* ATCC27343 (NC_007633), and 14232 (JFDO00000000.1), *M. capricolum subsp. capripneumoniae* 87001 and 99108 (CP006959, NZ_JMJI00000000.1), *M. leachii* PG50 (CP002108), *M. putrefaciens* KS1 and 9231 (CP003021, CP004357) and *M. yeatsii* GM274B and 13926 (CP007520, NZ_AORK00000000) were retrieved from GenBank to be used in phylogeny reconstruction. For *M. leachii* for which a single sequenced genome is available *in silico*, we added an experimental sequence obtained from a French isolate, namely *M. leachii* 06049.

### Pulse field gel electrophoresis (PFGE) protocol

PFGE analyses were conducted as described previously (Tardy et al., 2007). In brief, mycoplasma cells from overnight cultures were embedded in low melt agarose plugs and lysed by Proteinase K before DNA overnight restriction using endonucleases. The macrorestriction fragments were separated by electrophoresis on a CHEF-DR III system (Bio-Rad) in 1% agarose gel, in TBE 0.5% at 14°C, for 24 h, with an included angle of 120°. After testing six endonucleases (*Avi*II, *Bam*HI, *Nco*I, *Mlu*I, *Sma*I, and *Stu*I) for their discrimination capacity on genomic DNA from strain G5847^T^ and two randomly chosen French isolates, *Bam*HI and *Stu*I gave the highest number of bands and were selected. Images were analyzed with the software GelCompar II® v6.6 created by Applied Maths NV. The similarity analysis was carried out using the Dice coefficient (position tolerance, 1%) and a dendrogram was constructed by UPGMA method.

### *In silico* analyses

BlastP analysis was applied to search for putative enzymes involved in polysaccharide synthesis and described in other mycoplasma species (Bertin et al., 2013, 2015; Gaurivaud et al., 2016), in the Molligen database (http://services.cbib.u-bordeaux.fr/molligen/) using a 40% protein identity cut off (Barre et al., 2004). A search for secondary structures of predicted glycosyltransferases was carried out by TMHMM2 and InterProScan (Moller et al., 2001; Jones et al., 2014) and the synthase-specific DXD as well as R/QXXRW-like motifs were identified by alignment with the galactan synthase MSC_0108 from *Mmm* PG1^T^.

### Capsular polysaccharide extraction, purification, quantification, composition, and structure

Capsular polysaccharide (CPS) extraction and purification were performed as described previously (Gaurivaud et al., 2016). CPS were prepared from either 15 ml of exponential-phase cultures (*T* = 8 h, as estimated by growth kinetics in PPLO broth) or 10 ml of stationary-phase cultures (*T* = 24 h, as estimated by growth kinetics in PPLO broth) for dot-blotting experiments or from 145 ml stationary-phase cultures (24 h) for structure determination. The total sugars concentration in CPS was estimated by phenol/sulfuric acid method (Dubois et al., 1956), done in triplicate. The results are expressed as μg of glucose/ml of culture.

Dot-blotting experiments were performed as described previously (Bertin et al., 2015; Gaurivaud et al., 2016). In brief, 2 μl (*T* = 8 h) or 1 μg (*T* = 24 h) of purified CPS in a total volume of 2 μl were spotted onto a nitrocellulose membrane. Each dot was either stained using a polysaccharide detection kit based on Periodic Acid Schiff (PAS) staining (Glycoprotein detection Kit, Sigma-Aldrich) or revealed with anti galactan and anti β-(1→6)-glucan sera (Bertin et al., 2015; Gaurivaud et al., 2016).

To determine the structure of CPS from strains G5847^T^ and L15568, the first CPS purification steps were followed by a dialysis against regularly renewed ultrapure sterile water for 48 h at room temperature using 3.5-kDa-cutoff dialysis tubing (Spectrum Laboratories) to eliminate potential contaminants, and dried under vacuum. The monosaccharide components were determined after hydrolysis of CPS (1 mg) with 4 M CF_3_CO_2_H (100°C, 4 h). Aliquots of the extract were analyzed by high-performance anion exchange chromatography (HPAEC) equipped with a pulsed amperometric detector (Dionex ICS 3,000 system) and 4 × 50 mm Propac PA1 pre-column (Dionex) followed by a CarboPak PA 1 column at 30°C. Gradient elution was performed with a multi-step as follows: 0–25 min, 90% H_2_O and 10% NaOH 160 mM; 25–34 min, 100% NaOH 200 mM; 35–50 min 90% H_2_O and 10% NaOH 160 mM at a flow rate of 1 ml/min. Peak analysis was performed using Chromeleon software, version 7.0. CPS was dissolved in 99.96% D_2_O (1 mg/0.5 ml). Spectra were recorded, at 50°C, on a Bruker Avance 500 spectrometer equipped with a 5 mm BBI probe and Topspin 3.2 software. ^1^H NMR spectra were accumulated with water suppression by excitation sculpting with a gradients sequence provided by Bruker. ^13^C NMR experiments were conducted at 125.48 MHz with 2 s as relaxation delay. The 2D ^1^H/^1^H COSY, ^1^H/^1^H TOCSY, ^1^H/^1^H NOESY, ^1^H/^13^C HSQC, and ^1^H/^13^C HMBC spectra were acquired with standard pulse sequences delivered by Bruker.

## Results

### First report of *M. feriruminatoris* isolates in wild fauna in France and Italy

The *fusA* sequence of all putative *Mmc* isolates from Alpine ibex was generated to identify the isolates at the species level through phylogenetic reconstruction using the leBIBI^QBPP^ software (Flandrois et al., 2015). The closest sequence based on patristic distance for each isolate was unambiguously *M. feriruminatoris* 8756-C13. Thus, the 27 isolates from caprine ibex included in this study belong to the *M. feriruminatoris* species and not to *Mmc*, as originally thought based on MF-dot profiles. This is the first report of *M. feriruminatoris* presence in wild Alpine ibex in France. We also demonstrated that a previously unidentified isolate collected in Valle d'Aosta (Italy) from an Alpine ibex with severe conjunctivitis, namely 283F08 (Giangaspero et al., 2010), belongs to the *M. feriruminatoris* species on the basis of its *fusA* sequence.

### Phylogenetic positioning of *M. feriruminatoris* within the *M. mycoides* cluster

**Figure 1:**
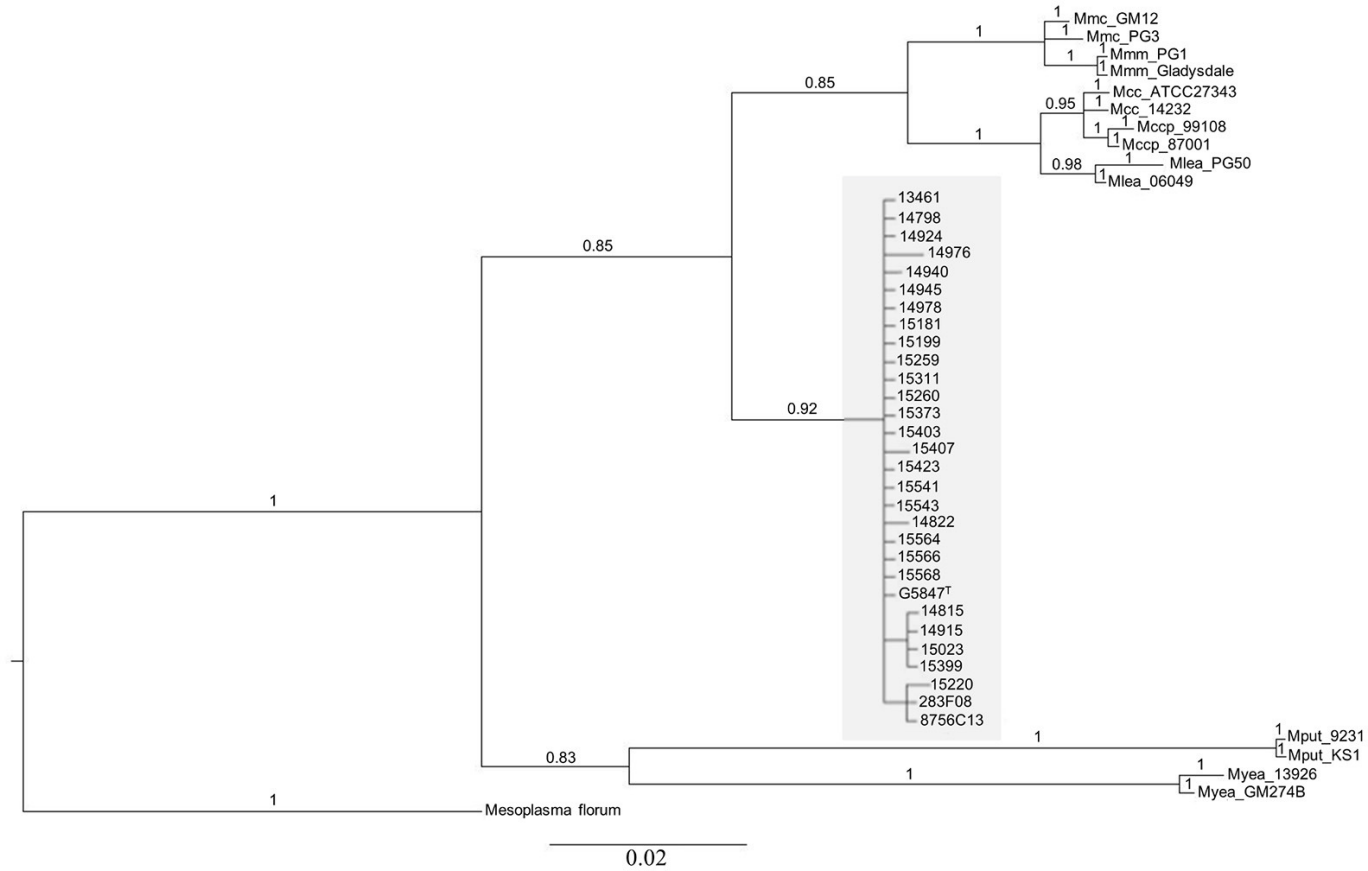
**Phylogenetic relationships of mycoplasma isolates, of which 28 represent the new species *M*. *feriruminatoris*, based on Bayesian analysis of 187 amino-acids alignment of the fusA protein**. The statistical supports indicated above branches correspond to Bayesian posterior probabilities. The scale bar below the tree shows the average number of substitutions per site. *M. feriruminatoris* isolates are shown in a gray box.

The position of *M. feriruminatoris* isolates with respect to the *M. mycoides* cluster was refined by running two phylogenetic analyses. The first one used a 187 amino acids subsequence of *fusA*, as previously described (Manso-Silvan et al., 2007), but involved a Bayesian approach. *Mesoplasma florum* was chosen as an outgroup, and several representatives of the different subspecies of the *M. mycoides* cluster, for which the genome has been published, were included (Figure 1). All *M. feriruminatoris* isolates grouped together in the same branch, the nearest group including all mycoplasmas from the *M. mycoides* cluster. This tree topology not only confirmed the identification of the isolates (all gathered with the type strain) but also clearly indicates that *M. feriruminatoris* belongs to the *M. mycoides* cluster with a high statistical branch support >0.85. The *M. feriruminatoris* branch is furthermore equidistant from the two *M. mycoides* subclusters, grouping the two *M. mycoides* subspecies on one side and the two *M. capricolum* subspecies on the other, as described previously (Manso-Silvan et al., 2007).

The 2nd phylogeny was reconstructed using the seven concatenated nucleotide sequences from the MLST scheme of Fischer et al., 2012, *i.e., rpoB, pgI, pdhC, gyrB, gmk, adk*, and *recA*. The Bayesian analysis parameters, the outgroup and other reference strains were the same as in the *fusA* phylogeny. The overall topology of the tree (Supplementary Figure 1) was comparable to that in Figure 1, all *M. feriruminatoris* isolates being grouped in a single branch, the nearest neighbor being the branch corresponding to the *M. mycoides* cluster, with strong Bayesian posterior probability for each branch. This result confirms their belonging to the *M. mycoides* cluster *sensu stricto* unlike *M. putrefaciens* and *M. yeatsii*. These two last species are responsible for the only divergence between the two trees topologies as they are grouped on a common branch based on the fusA amino acid sequence but split on two individual branches according to Fisher's MLST scheme. Nonetheless, both phylogenies indicate that *M. putrefaciens* and *M. yeatsii* are clearly not in the *M. mycoides* cluster while *M. feriruminatoris* might constitute the 4th species of the cluster (which previously included *M. mycoides, M. capricolum* and *M. leachii* species). Furthermore, this phylogenetic study not only confirmed that the French isolates from ibex belong to the *M. feriruminatoris* species but also illustrated their important overall evolutionary relatedness with very short branches, whether they originated from France, Italy, USA or Germany.

### Absence of clonality between isolates from French alpine ibex

**Figure 2:**
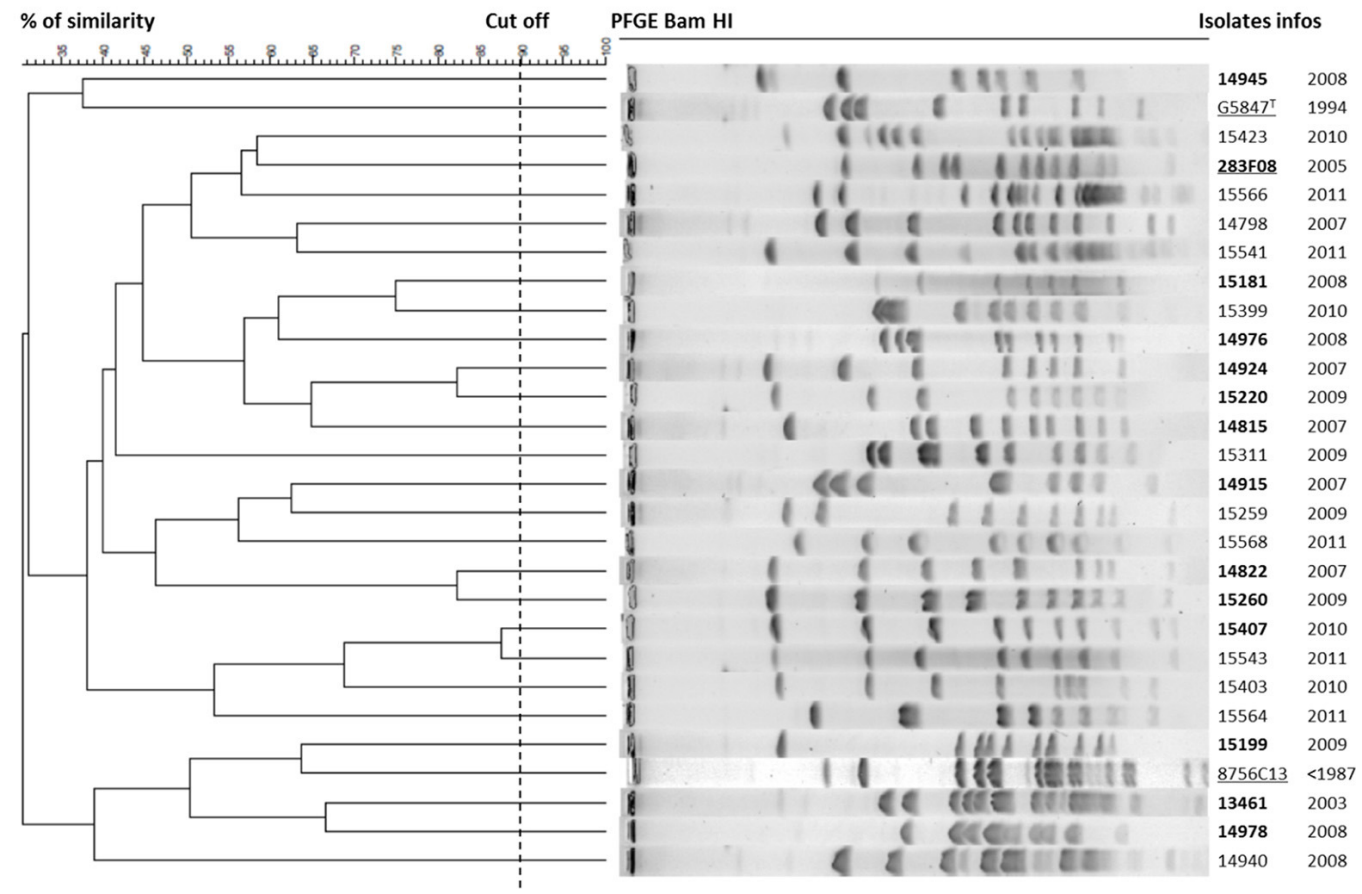
**Dendrogram displaying *Bam*HI-PFGE profiles**. Cluster analysis of 28 *M. feriruminatoris* isolates based on their PFGE-*Bam*HI profiles using the Dice coefficient and UPGMA. The resulting degree of similarity is indicated on the scale on the left. *M. feriruminatoris* isolates are characterized by their collection number and year of isolation. Reference strains are underlined and isolates collected from ibex with clinical signs are in bold type.

As both *fusA*-locus sequence typing and MLST failed to discriminate the different *M. feriruminatoris* isolates, with only a few polymorphisms in housekeeping genes observed (Figure 1, Supplementary Figure S1) we further attempted to investigate their relatedness using a genomic macrorestriction approach. *Bam*HI and *Stu*I enzymes were selected as, of all the enzymes tested on a set of three strains, they yielded the most interpretable and discriminatory banding patterns (data not shown). Each of our isolates presented a unique restriction pattern and two isolates (L15373 and L15023) were removed from the analysis because they could not be typed. Between two to seven bands and seven to thirteen bands were generated by *Stu*I (data not shown) and *Bam*HI (Figure 2), respectively. All the isolates were different (percentage of global similarity <90%) whatever the associated clinical signs, sampling year or potential co-isolation with other mycoplasmas (see Table 1 and Figure 2). As the number of bands was less than that recommended by Tenover et al. (1995), each isolate was reanalyzed on a global restriction pattern using both endonucleases (data not shown) with GelCompar II software which calculates the global similarity of isolates by merging multiple electrophoreses fingerprints. Again, all the isolates were different and none could be grouped together. For instance isolates L15407 and L15543 that shared a similar banding pattern with *Bam*HI (Figure 2) were remote in the dendrogram generated with the 2 restriction enzymes (data not shown). Furthermore, the *M. feriruminatoris* G5847^T^, 283F08 and 8656-C13 profiles were randomly distributed amongst French isolates (Figure 2). These results suggest that the French isolates of *M. feriruminatoris* collected between 2003 and 2011 in a restricted region of the Alps and from a homogeneous ibex population were not clonal. Their banding patterns are unique and do not present greater similarities than the banding patterns of *M. feriruminatoris* isolates collected elsewhere in the world (Italy, USA and Germany).

### Design and validation of a *M. feriruminatoris* specific PCR assay

A specific PCR assay was developed to allow rapid and accurate discrimination of this new member of the *M. mycoides* cluster, for which both PCR/DGGE and MF-dot identification had failed (Giangaspero et al., 2010; Tardy et al., 2012). DNA sequences putatively specific to G5847^T^ were identified in the Molligen database using the “Comparative Differential query” Toolbox. The bidirectional best hit (BDBH) algorithm indicated the presence of 66 partial or complete CDS in the contigs of *M. feriruminatoris* G5847^T^, which were absent from the genomes of strains belonging to the *M. mycoides* cluster and from *M. putrefaciens* and *M. yeatsii*. Amongst these 66 candidate genes, all those (i) with a size smaller than 300 nt, not suitable for an end-point PCR design (*n* = 30), or (ii) corresponding to partial CDS (truncated genes at the contig extremities, *n* = 12), or (iii) encoding putative lipoproteins, membrane proteins and restriction-modification enzymes (*n* = 20), that are very common in mycoplasmas, were discarded. One of the remaining 4 genes, D500_0409 was further eliminated as its product showed more than 50% amino acid identity with *M. agalactiae*, a species potentially co-isolated with *M. feriruminatoris*. Of the 3 remaining hypothetical proteins, the shortest genes (D500_0723 and D500_0126) were excluded and the 1134 bp- long D500_0332 gene was selected as a candidate for PCR assay. This gene codes for a protein of unknown function but harboring two domains, a N-terminal “Putative DNA-binding” domain (PFAM04326) and a C-terminal “Putative ATP-dependent DNA helicase recG” domain (PFAM13749). Two primers were designed to amplify a 765 pb locus that includes the “Putative DNA-binding” domain. PCR assays were performed using isolates of *M. feriruminatoris* and strains belonging to the *M. mycoides* cluster, *M. putrefaciens, M. yeatsii, M. bovis, M. agalactiae, M. ovipneumoniae, M. conjunctivae*, and *M. arginini*. As expected, results were exclusively positive for all *M. feriruminatoris* strains and no amplification was observed for any of the other species tested (Supplementary Figure S2). The limit of detection of D500_0332 specific PCR was tested using 10-fold dilutions of G5847 DNA extract from 3.5 × 10^9^ CFU/ml liquid culture. Amplicons were obtained up to a dilution of 10^−4^ which corresponds to a limit of detection of 0.02 ng/μl of DNA per reaction and is equivalent to 3.5 × 10^2^ CFU per reaction based on a DNA extraction yield of 100%.

### Capsular polysaccharides production by *M. feriruminatoris* G5847^T^

Despite being closely related from a phylogenetic point of view, (sub)species of the *M. mycoides* cluster have been shown to differ in terms of polysaccharide production (Bertin et al., 2013, 2015; Gaurivaud et al., 2016). We therefore investigated whether *M. feriruminatoris* was able to produce capsular polysaccharides and the nature of these polysaccharides.

**Figure 3:**
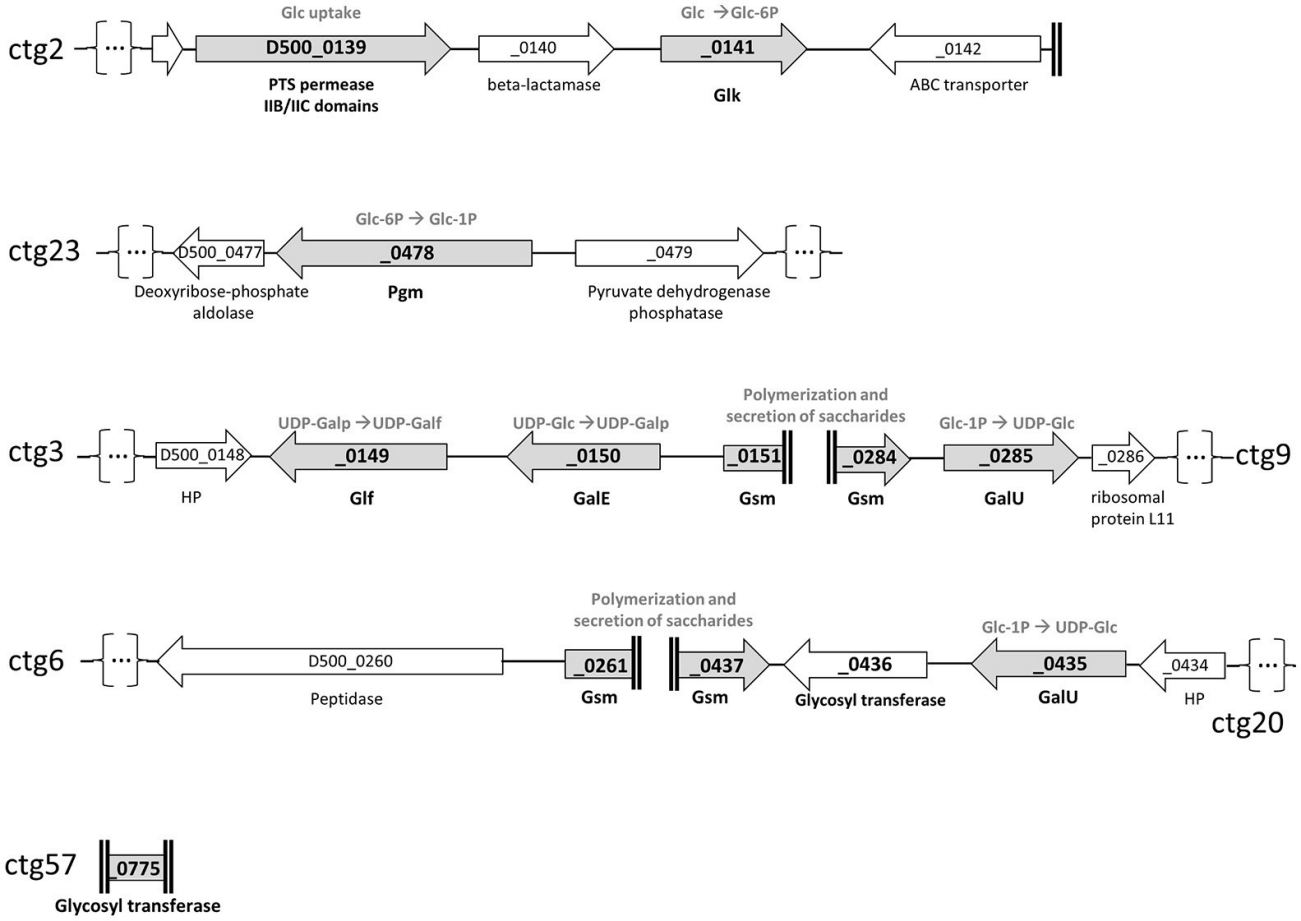
**Schematic representation of genome loci involved in polysaccharides biosynthetic pathways as predicted from the genome of *M*. *feriruminatoris* sp. nov. strain G5847^T^**. The coding sequences (CDS) are represented by arrows and designated by their locus tags. The name of the contigs (ctg) from which the CDS were retrieved are indicated on the right or the left of the figure. Ends of contigs are represented by double vertical bold lines and contig interruption, for representation purposes, by dots into brackets. Gray arrows correspond to CDS potentially involved in polysaccharide biosynthesis. These are a phosphotransferase system (PTS)-glucose permease and a glucokinase (Glk) for glucose (Glc) uptake and phosphorylation into glucose-6-phosphate; a phosphoglucomutase (Pgm) for isomerization of the glucose-6-phosphate into glucose-1-phosphate (Glc-1P); a glucose-1-phosphate uridylyltransferase (GalU) to transform Glc-1P to UDP-glucose; an UDP-glucose 4-epimerase (GalE) and an UDP-galactofuranose mutase (Glf) to successively transform the UDP-glucose into UDP-galactofuranose; a (or several) glycosyltransferase (GT) with synthase activity (Gsm; Glycan synthase of mollicutes) to build and export the final polysaccharide, suspected to be a polymer of galactofuranose (galactan). HP, Hypothetical Protein.

Firstly, the genome of *M. feriruminatoris* G5847^T^, available *in silico* as 92 contigs (Fischer et al., 2013) was searched for homologs of enzymes involved in polysaccharides biosynthesis, as predicted in several species of the *M. mycoides* cluster and *M. agalactiae* (Bertin et al., 2013, 2015; Gaurivaud et al., 2016). BlastP analyses allowed retrieval of a complete putative biosynthetic pathway leading to galactan production, thus confirming or supplementing data from genome annotation (Figure 3). Contig2 carries genes involved both in glucose uptake (phosphotransferase system (PTS)-glucose permease, D500–0139) and glucose phosphorylation (glucokinase, *Glk*, D500_0141). Genes encoding enzymes leading to the isomerization of phosphorylated glucose (phosphoglucomutase, *Pgm*, D500_0478) and its activation as a UDP sugar (glucose-1-phosphate uridylyltransferase, *GalU*, D500_0285) were identified on contig23, and contig9 as well as contig20, respectively. The presence of a putative UDP-glucose 4-epimerase (*GalE*, D500_0150) and an UDP-galactofuranose mutase (*Glf*, D500_0149) on contig3 suggested that UDP-glucose can further be transformed into UDP-galactofuranose. Several truncated genes were predicted to encode glycosyltransferases and found to display structural similarity to the previously described glycan synthase (Gsm) encoded by *M. mycoides* subsp. *mycoides* PG1^T^, MSC_108 (Gaurivaud et al., 2016). These were D500_0151 (end of contig3), D500_0261 (contig6), D500_0284 (contig9), D500_0437 (contig20), and D500_0775 (whole contig57).

To close the genomic gaps and generate a continuous sequence for the synthases, several PCR-assays using pair-wise combinations of primers picked upstream and downstream from the different genes were designed (see Table 2). The sequence analysis of the resulting PCR products revealed two complete synthase sequences located in contig3 and contig9 (hereafter called Gsm3-9) and in contig6 and 20 (called Gsm6-20), respectively. Because the D500_0775 partial sequence (521 nt), was restricted to the GT2 glycosyltransferase domain we were unable to further analyze its function and genomic position. Gsm3-9 and Gsm6-20 are of approximately the same length (1,380 vs. 1,383 bp) and show a conserved sequence (with 88.07% identity in nt), suggesting potential gene duplication. A search for structural similarities with previous described synthases was then carried out using TMHMM2 and InterProScan (Moller et al., 2001; Jones et al., 2014). Both synthases displayed 4 transmembrane domains (TMDs) and a cytoplasmic domain with DXD and R/QXXRW motifs of the GT-A glycosyltransferase family (Breton et al., 2006). More precisely their cytoplasmic domain motifs, DAD and QRMRW, and the presence of 4 TMDs designate them as galactan synthases, homologs to that encoded by MSC_0108 in *M. mycoides* subsp. *mycoides* PG1^T^ (Gaurivaud et al., 2016). Thus, *in silico* analyses are strongly in favor of G5847^T^ being able to produce galactan via a synthase dependent pathway.

**Figure 4:**
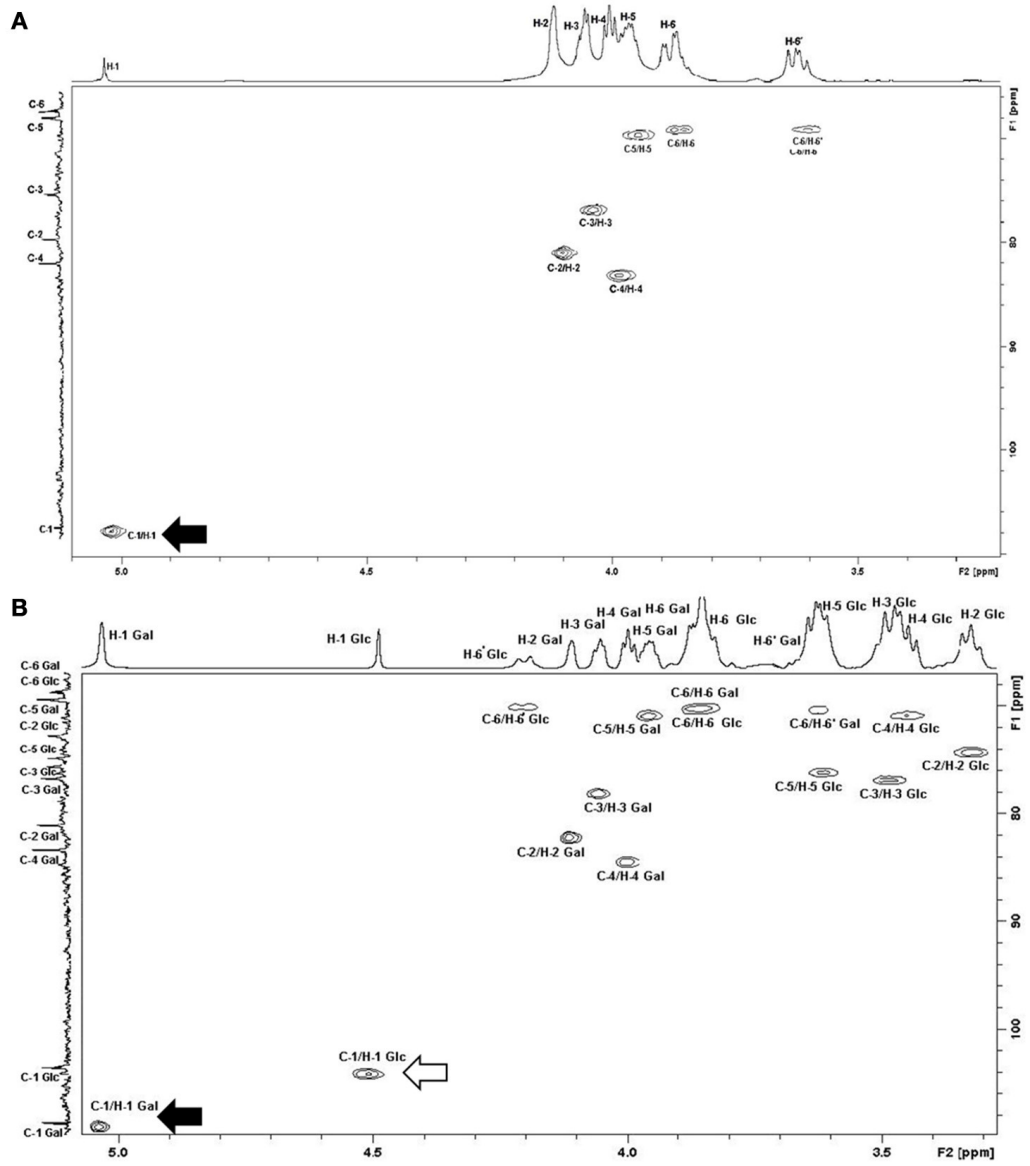
**^1^H/^13^C HSQC NMR spectra of CPS from (A)** *M. feriruminatoris* G5847^T^; **(B)** *M*. *feriruminatoris* 15568. The H-1 signal at 5 ppm and that of C-1 at 109 ppm were typical of D-galactose residues with β-furanoside configuration (black arrow). The chemical shift value obtained for C-6 indicated the presence of a linkage on the C-6 galactan residue. Thus, both strains G5847^T^ and 15568 are β-(1→6)-galactan producers. The H-1 signal at 4.5 ppm and that of C-1 at 104 ppm were typical of D-glucose residues with β-pyranoside configuration (white arrow). The H-6/C-6′ chemical values indicated the presence of a linkage on the C-6 glucose residue. These values confirm that the 15668 isolate produces both β-(1→6)-galactan and β-(1→6)-glucan.

We further investigated the hypothesis of galactan synthesis *in vitro* by quantifying and purifying *M. feriruminatoris* G5847^T^ capsular polysaccharides (CPS) after a 24 h growth culture. HPAEC with Pulsed Amperometric Detection confirmed that the CPS produced by *M. feriruminatoris* G5847^T^ at 24 h was composed solely of galactose. ^1^H NMR spectra of purified CPS further showed 7 resonances (Figure 4A) and the correlations observed in 2D NMR COSY and HSQC spectra enabled us to attribute the various chemical shifts of galactosyl residues: H-1/C-1 (5.046/109.0 ppm), H-2/C-2 (4.118/82.1 ppm), H-3/C-3 (4.068/78.1 ppm), H-4/C-4 (4.013/84.5 ppm), H-5/C-5 (3.965/70.8 ppm) and H-6–H-6′/C-6 (3.864;3.648/70.2 ppm). The ^13^C signal at 109.0 ppm and ^1^H signal at 5.046 ppm were typical of D-galactose residues with a β furanoside configuration (Bertin et al., 2013). The ^1^H/^1^H NOESY spectrum and the ^1^H/^13^C HMBC spectrum presented connectivities between the H-1/C-1 of galactose and the H-6–H-6′/C-6 of galactose. Thus, the CPS produced by *M. feriruminatoris* G5847^T^ at 24 h was indeed a β-(1→6)-galactofuranose homopolymer, commonly named galactan, as predicted from *in silico* analysis.

### Diversity of capsular polysaccharides produced by different *M. feriruminatoris* isolates

**Figure 5:**
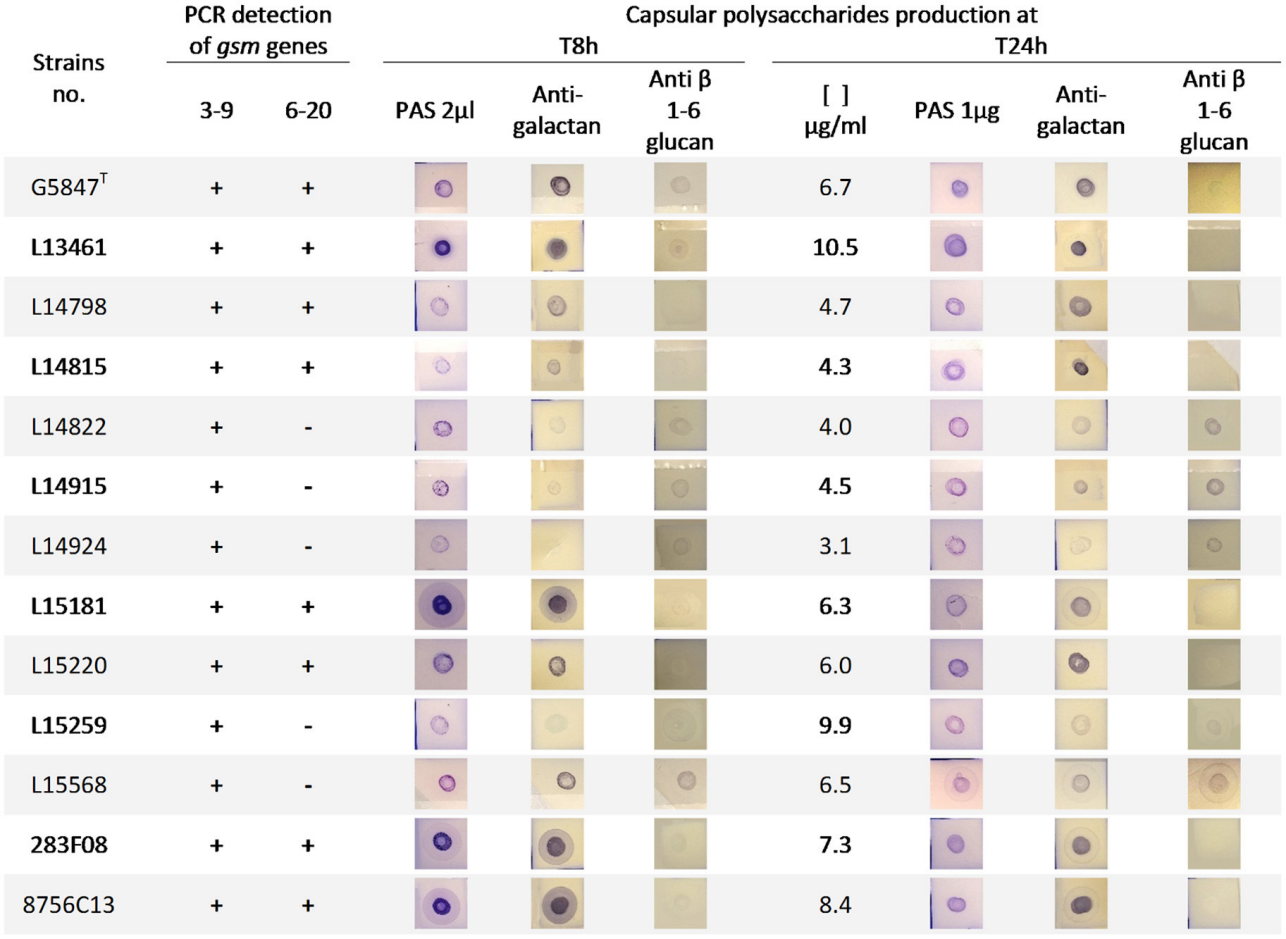
**Presence of *gsm* genes and capsular polysaccharides production in *M*. *feriruminatoris* isolates**. *Gsm* genes identified in the type strain G5847, namely gsm3-9 and gsm6-20, were detected (+) or not (−) by two specific PCRs. Capsular polysaccharide (CPS) were purified from 8 to 24 h cultures and identified by dot-blots revealed by Periodic acid–Schiff (PAS) staining (general polysaccharides detection) and antisera targeting galactan and anti β-(1→6)-glucan. At 24 h, the purified CPS were further quantified (μg of glucose equivalent per ml of culture) and 1 μg was deposited on the nitrocellulose membrane.

In order to decipher whether the type strain G5847^T^ was representative of other *M. feriruminatoris* sp. nov. isolates in terms of CPS production we performed a dot blot analysis on a subset of 10 isolates, randomly selected from our French population, as well as on strains 283F08 and 8756-C13 (Figure 5). CPS were purified at both 8 and 24 h to assess their presence in the exponential vs. stationary growth phase. However, they were quantified only at 24 h because of the expected low level of production at 8 h due to poor cell counts. Blots were revealed using PAS staining for semiquantitative detection of polysaccharides and antisera targeting galactan as well as β-(1→6)-glucans, two polysaccharides produced by *M. mycoides* subspecies (Bertin et al., 2015; Gaurivaud et al., 2016), the closest taxon of *M. feriruminatoris* (Figure 5). β-(1→2)-glucan was not tested in the present set of strains because previous preliminary results obtained with 4 different French isolates had not yielded any positive dots (data not shown), suggesting that β-(1→2) glucan is not produced by *M. feriruminatoris* isolates.

All isolates were able to produce galactan as a CPS both in exponential and stationary phases, like the type strain G5847^T^. This is in agreement with the presence of a gene putatively encoding the Gsm3-9 synthase as detected by PCR for all isolates (Figure 5). In contrast PCRs targeting the Gsm6-20 synthase gave no amplification in 5 out of 12 isolates suggesting that it might play a minor role in the overall CPS production. Both PAS staining at 8 h and bulk glucose quantification at 24 h (Figure 5) illustrated the different individual capacities of strains to produce CPS, from 3.1 to 10.5 μg per ml of culture. This was apparently not associated with the presence of a second Gsm as strain L15259 produced a lot of CPS (9.9 μg/ml) but was PCR negative for the Gsm6-20 gene.

Surprisingly, antibodies raised against β-(1→6)-glucopyranose reacted with purified CPS of different strains, at 8 and 24 h, although to a lesser extent and with sometimes negative or close-to-the-limit-of-detection spots. This result suggested the potential synthesis of β-(1→6)-glucans, as a second component of CPS in several isolates.

To confirm this hypothesis, CPS of strain L15568 which showed strong immunostaining with both antibodies raised against galactan and β-(1→6)-glucan were analyzed by HPAEC and NMR spectra. HPAEC showed that the CPS purified from strain L15568 at 24 h consisted mainly of galactose but also of glucose with a 2:1 ratio. The ^1^H NMR spectra revealed a signal corresponding to the β-(1→6)-galactofuranose homopolymer like the CPS from G5847^T^ (Figure 4B), but also a number of additional signals due to the presence of glucose. Based on correlations observed on the 2D NMR COSY spectrum, chemical shifts to the proton of glucosyl residues could be attributed: 4.511 (H-1), 3.325 (H-2), 3.487 (H-3), 3.448 (H-4), 3.617 (H-5), 3.852 (H-6), and 4.202 (H-6′) ppm. On the 2D NMR HSQC spectrum (Figure 4B), the connectivities observed between H-1 and C-1 (104.0 ppm), H-2/C-2 (74.1 ppm), H-3/C-3 (76.8 ppm), H-4/C-4 (70.7 ppm), H-5/C-5 (76.2 ppm) and H-6, H-6′/C-6 (70.1 ppm) were characteristic of a glucan homopolymer. The H-1 signal at 4.51 ppm (^1,2^*J* = 7.84 Hz) and C-1 at 104 ppm were typical of D-glucose residues with β pyranoside configuration (Gaurivaud et al., 2016). H-6′ chemical values indicated the presence of a linkage on the C-6 in glucose residues. In the ^1^H/^13^C HMBC spectrum, the absence of connectivities between the H-1 of galactose and carbon of glucose, and between the H-1 of glucose and carbon of galactose, demonstrated the presence of two homopolymers, and not one heteropolymer of glucose and galactose. Hence, the CPS from *M. feriruminatoris* L15568 was composed of two homopolymers: mainly a β-(1→6)-galactan and also a β-(1→6) glucan. This is the first time that two different CPS have been identified in a single mycoplasma isolate belonging to the *M. mycoides* cluster.

The gene(s) involved in β-(1→6)-glucan production have yet to be identified. In the genome of G5847^T^, a PCR screening for the presence of β-(1→6)-glucan Gsm using primers designed on *Gsm* genes from *M. agalactiae* 14628 and *Mmc* PG3^T^ strains that are β-(1→6)-glucan producers was unsuccessful (data not shown). However, the dot blot resulting from CPS extraction after 8 h-growth of strain G5847^T^ clearly gave a positive reaction with antisera directed against β-(1→6)-glucan. At 24 h, β-(1→6)-glucan production seems to decrease which explains why it was not detected in our NMR analysis.

These results demonstrate that *M. feriruminatoris* sp. nov. strains are able to produce both attached galactan and β-(1→6)-glucan polymers and that the level of synthesis of β-(1→6)-glucan might vary in time and in environmental conditions.

## Discussion

The first part of the the study was carried out to refine the identification of mycoplasma strains isolated from *Capra ibex* in the French Alps and originally classified as *Mmc* on the basis of their antigenic profile (Tardy et al., 2012). Sequence analysis of *fusA* and several other housekeeping genes revealed that these strains actually belong to the newly proposed *M. feriruminatoris* species, of which this is the first report in France. The previously unidentified 283F08 Italian strain (Giangaspero et al., 2010) was also assigned to the *M. feriruminatoris* species. These recent (between 2003 and 2011) isolations contrast with the reference strains collected long ago (before 1987 and 1993 for 8756-C13 and G5847^T^, respectively) and confirm the species status of the *M. feriruminatoris* taxon. The French isolates might share a common origin with the Italian isolate 283F08 as the ibex vital area overlaps the two sampling sites. Nonetheless, this is the first report of *M. feriruminatoris* in a wild population of Alpine ibex, as the G5847^T^ strain was collected in a zoo in Berlin (Germany). To improve our knowledge of the geographical distribution of the *M. feriruminatoris* species, it would be interesting to re-analyse all unassigned *M. species* strains described so far in wild ruminants worldwide (Nicolas et al., 2005; Gonzalez-Candela et al., 2007). As MF-dot signature, growth inhibition tests and 16S rDNA DGGE analysis all failed to unambiguously identify *M. feriruminatoris* isolates (Manso-Silvan et al., 2007; Giangaspero et al., 2010; Tardy et al., 2012), we developed a specific PCR assay targeting the D500_0332 gene shown *in silico* to be present only in *M. feriruminatoris* and in no other *Mycoplasma* species. This assay is suggested as an alternative routine tool, less time-consuming than sequence analysis of the *fusA* gene, to improve the capacity of laboratories to detect and identify new *M. feriruminatoris* isolates.

The genetic diversity of *M. feriruminatoris* species was previously estimated to be very low based on polymorphisms in housekeeping genes. However, this analysis was run in only 5 isolates, 4 of which were collected in the same zoo within <2 years (Fischer et al., 2012). Here our PFGE analysis was able to discriminate all the individual isolates whatever their origin. Hence, the French isolates were not clonal despite their collection from a restricted area in the Alps and from a homogeneous ibex population. They diverged as much between each other as between isolates from other sources (USA, Germany zoo and Italy). Therefore, we considered that the *M. feriruminatoris* taxon was a true species, with few polymorphisms in housekeeping genes between strains, as shown in Supplementary Figure S1, but with considerable variability in their genomic macrorestriction profiles, as already demonstrated for the close *Mmc* subspecies (Tardy et al., 2007).

Another major outcome of this study was to propose *M. feriruminatoris* as a fourth species within the *M. mycoides* cluster. The *M. mycoides* cluster is a phenotypically and genetically cohesive group of 5 (sub)species, gathered into 3 species, namely *M. capricolum, M. mycoides* and *M. leachii*, that are all pathogenic for ruminants. The cluster has an eccentric phylogenetic position within the family *Entomoplasmataceae*, which includes species associated with arthropod or plant hosts. This taxonomic anomaly is due to the fact that the discovery and naming of *Mycoplasma mycoides*, the etiological agent of CBPP, preceded the establishment of the *Entomoplasmatales* order by a century (Brown, 2010). Genetic relationships between the (sub)species within the cluster have been extensively studied in recent decades but few studies have been dedicated to the positioning of *M. feriruminatoris* since its proposal as a new species (Weisburg et al., 1989; Pettersson et al., 1996; Manso-Silvan et al., 2007, 2009; Tardy et al., 2009; Fischer et al., 2012). Because of the lack of informative polymorphisms within the 16S rRNA gene sequences, alternative phylogenetic analyses were developed based on different sets of concatenated sequences from housekeeping genes (Manso-Silvan et al., 2007; Fischer et al., 2012). The first MLST scheme positioned the *M. feriruminatoris* strain 8756-C13 midway between the *M. mycoides* cluster and a group of related species with high 16S rRNA similarity (*M. yeatsii, M. cottewii* and *M. putrefaciens*), considered as non-core species of the cluster in its strict definition (Manso-Silvan et al., 2007). In the 2nd attempt, the *M. feriruminatoris* species (represented by 5 strains) was excluded from the phylogenetic study as its acceptability as an outgroup was jeopardized by substitution saturation in the sequences comparison. Because the 16S rDNA phylogeny of mycoplasmas clearly roots the *M. mycoides* cluster and other non-core species in the *Mesoplasma* species (Johansson and Pettersson, 2002; Breton et al., 2012), we proposed a new phylogenetic analysis using *Mesoplasma florum* as an outgroup and including several non-core species. All 30 *M. feriruminatoris* isolates included in the study were positioned in the main branch of the *M. mycoides* cluster while *M. putrefaciens* and *M. yeatsii* remained separated in remote branches. This strongly suggests that *M. feriruminatoris* might be a new species of the cluster, which is coherent with the fact that MF-dot failed to distinguish *M. feriruminatoris* from *Mmc* isolates (Tardy et al., 2012). These results raise the question about the date of divergence between *M. feriruminatoris* in wild ruminants and its co-evolution with the ibex host. The work of Fisher et al. dated the origin of the *M. mycoides* cluster back to a period concomitant with the domestication of small and large ruminants (Fischer et al., 2012). All members of the cluster had then become important pathogens, several of which being currently listed by the OIE. Whether *M. feriruminatoris* also emerged at the same time from a common ancestor and has evolved as a pathogen has yet to be demonstrated. So far there is no indication that *M. feriruminatoris* is a real pathogen in ibex or could represent a threat to the neighboring lifestock.

*Mycoplasma feriruminatoris* isolates were shown to produce at least two different capsular polysaccharides with relative proportions that varied in time and with environmental conditions. These were β-(1→6)-galactan and β-(1→6)-glucan homopolymers. We previously demonstrated that galactan synthesis in mycoplasmas of the *M. mycoides* cluster was associated with the presence of a 4 TMD synthase, of which the prototype is the product of gene MSC_0108 in *M. mycoides* subsp. *mycoides* PG1^T^, retrieved from several isolates of *Mmm* and *Mmc* serovar LC, while β-(1→6)-glucan production depended on the presence of a 7 TMD synthase retrieved from *Mmc* PG3^T^ and several isolates of *M. agalactiae*, of which the prototype is the product of gene MAGb_1260 in *M. agalactiae* 14628 (Bertin et al., 2015; Gaurivaud et al., 2016). Five loci in the genome of *M. feriruminatoris* G5847^T^ were identified as potentially coding synthases but as the genome had not been completed they were often interrupted by contig ends. By a PCR approach we were able to demonstrate the presence of two complete genes encoding 4 TMD glycosyltransferases with motifs characteristic of synthases that could account for galactan synthesis. In contrast no 7 TMD synthase was predicted from *in silico* data that could account for β-(1→6)-glucan synthesis. This suggests the existence of other, not yet described mechanisms for β-(1→6)-glucan synthesis and export in *M. feriruminatoris*. The D500_0436 locus, which harbors a family 2 glycosyltransferase domain and shows similarity with the MSC_0109 gene of *Mmm* PG1^T^ that was proposed to be a glycolipid synthase able to transfer galactosyl and glucosyl residues to membrane-bound diacylglycerol (Bertin et al., 2013) may also participate but this possibility has yet to be explored. *M. bovigenitalium* is the only mycoplasma species so far in which two synthases with different specificities have been predicted *in silico* (Gaurivaud et al., 2016). In another mycoplasma species, *M. pulmonis*, the type strain was shown to produce two types of capsular polysaccharides, EPS-I composed of glucose and galactose and EPS-II containing *N*-acetylglucosamine (Daubenspeck et al., 2009). Through mutation/complementation analysis, the production of EPS-I has been associated with the presence of a heterodimeric pair of ABC transporter permeases encoded by genes MYPU_7410 and 7420. No homologs of MYPU_7410 and 7420 have been identified in *M. feriruminatoris* G5847^T^ genome despite the presence of several other ABC transporters. These results strongly support the potential existence of several different pathways for capsule synthesis in mycoplasmas, including different anchoring systems and acceptor molecules.

The production of galactan by *Mmm, Mmc* serovar LC and *M. feriruminatoris* is another strong argument for the latter belonging to the cluster *M. mycoides*–and more specifically to the *M. mycoides* subcluster as no β-(1→2)-glucan, characteristic so far of the *M. capricolum* subcluster was detected- and may take part in the serological cross-reactions observed between these species in MF-dot. However, in contrast to other members of the *M. mycoides* cluster, the pathogenicity of *M. feriruminatoris* has not been established and, in the present study, most of the isolates originated from the ear canal of ibex with or without clinical signs. Hence, the role of capsular polysaccharides in *M. feriruminatoris* interaction with its ibex host has yet to be explored. Once again, this configuration could be compared to that of *Mmc*, with the coexistence of strains responsible for clinical outbreaks and other (very variable) strains recovered from the ear canal of asymptomatic, healthy goats (Tardy et al., 2007). The galactan capsule was shown to protect *Mmm* from serum activity while the β-(1→6)-glucan capsule of *M. agalactiae* enhances cell-killing through the serum complement (Gaurivaud et al., 2014, 2016). Phenotypic switching between different levels of production of one or the other polysaccharide can be a real asset to adapt to changing environments, an important feature in wild fauna hosts. Interestingly the previously described *M. agalactiae* strains from ibex showed lots of *M. mycoides*-like features and notably their capacity to produce a β-(1→6)-glucan capsule like *Mmc* PG3^T^ (Tardy et al., 2012; Gaurivaud et al., 2016). This could result from genetic exchange while sharing the same ibex hosts with *M. feriruminatoris*.

In conclusion, the present study contributes to better characterize the *M. feriruminatoris* new species and compare it with other members of the *M. mycoides* cluster. Because the French isolates are of recent origin it also confirms the current circulation of these mycoplasmas, which are not an evolutionary dead-end. Together with their genetic plasticity as shown by the macrorestriction patterns, this suggests that *M. feriruminatoris* might be a key player in the overall evolution of the genus and might help to untangle the *M. mycoides* cluster.

## Author contributions

FT conceived the work and carried out the initial analyses of the French isolates. CA, PG, YG, CP, and FT were involved in the acquisition and/or analysis of the data. CA and FT drafted the manuscript and produced the figures and tables.

### Conflict of interest statement

The authors declare that the research was conducted in the absence of any commercial or financial relationships that could be construed as a potential conflict of interest.
