# Supplementary figures and images for: Identification and Characterization of Mycoplasma feriruminatoris sp. nov. Strains Isolated from Alpine Ibex: A 4th Species in the Mycoplasma mycoides Cluster Hosted by Non-domesticated Ruminants?
Source: Front Microbiol. 2017 May 30;8:939. doi: 10.3389/fmicb.2017.00939 (PMC5447728; doi:10.3389/fmicb.2017.00939)

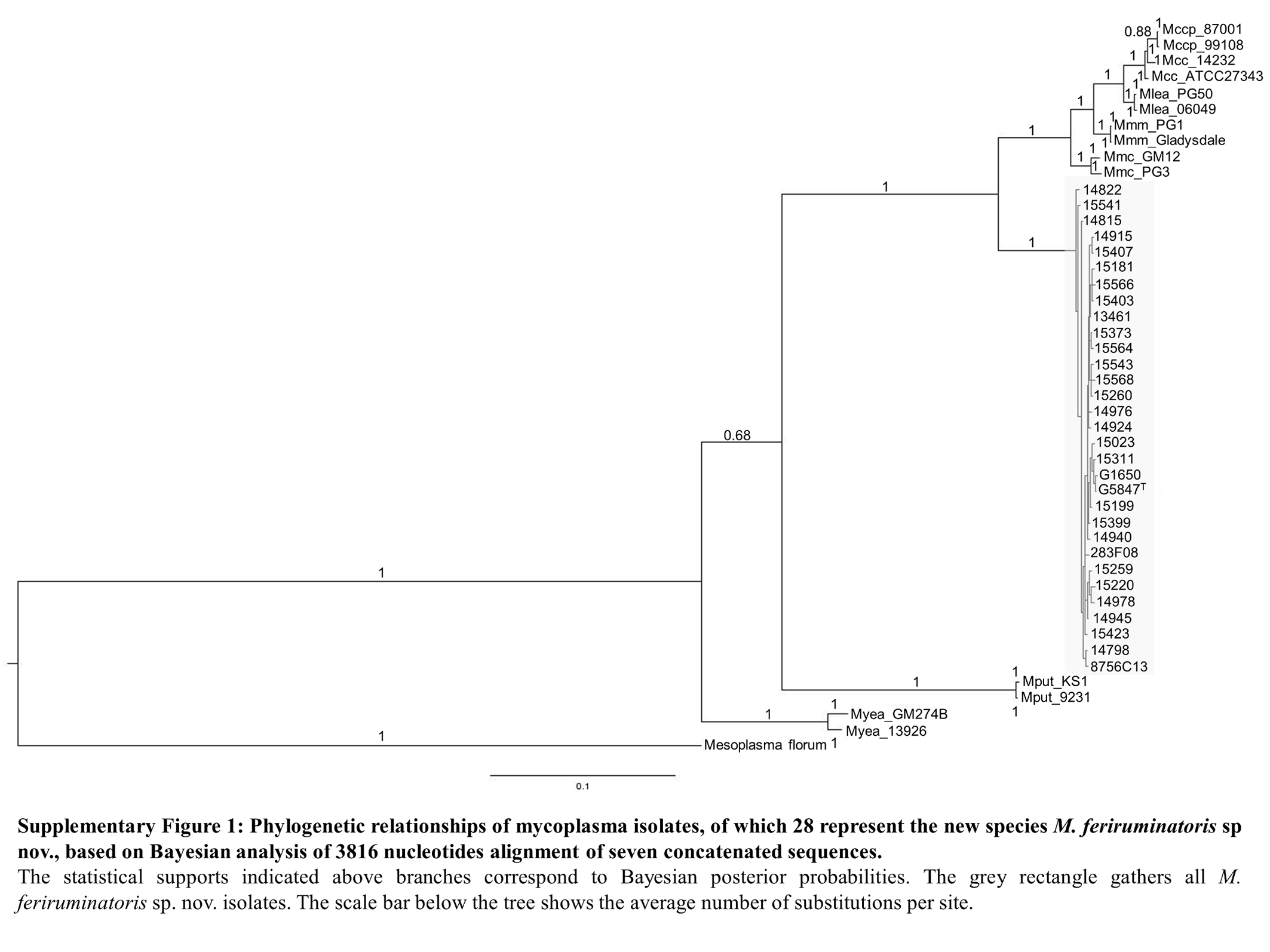

Supplement: Supplementary file 1 [file Image1.TIF]

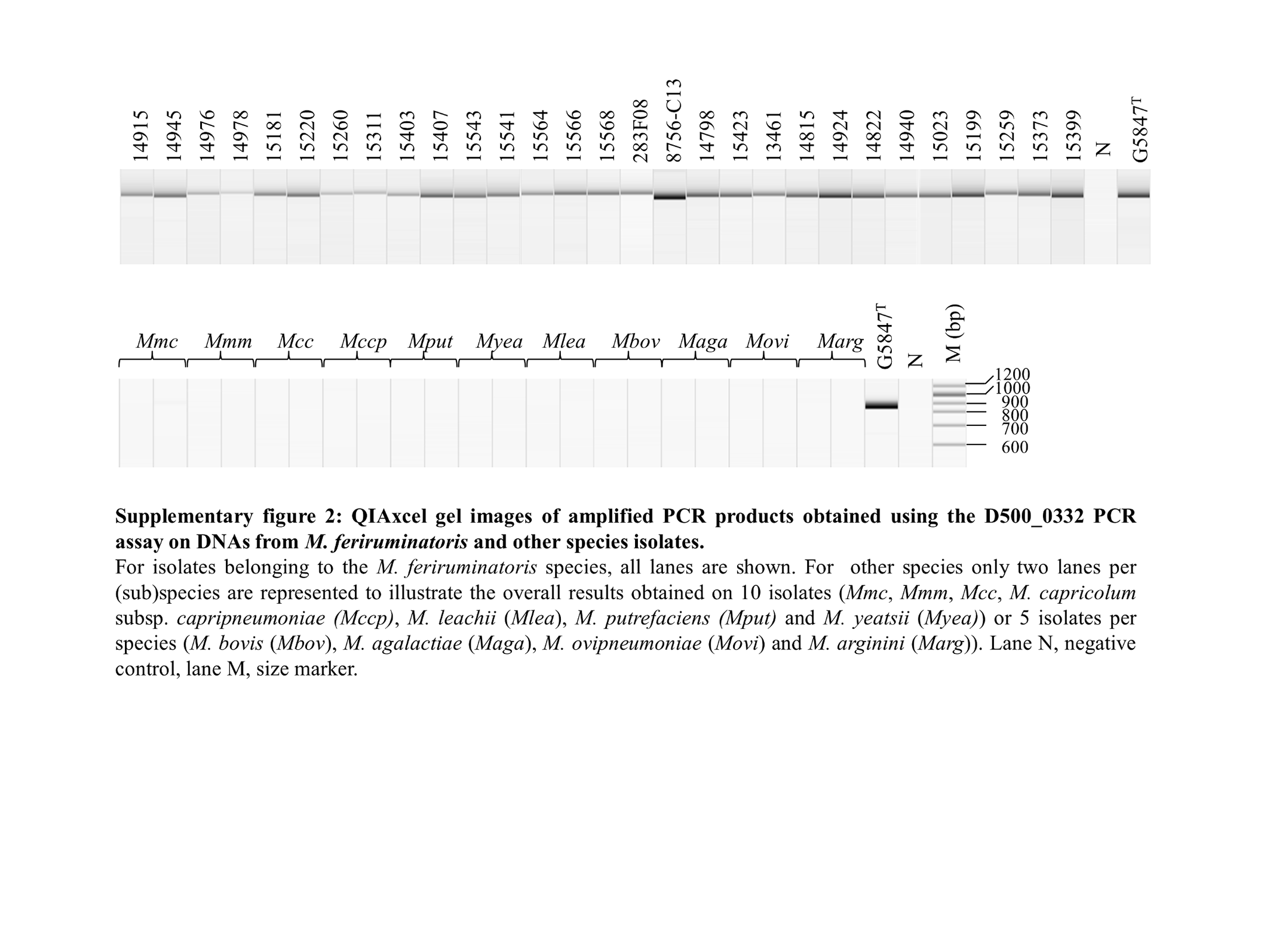

Supplement: Supplementary file 2 [file Image2.TIF]
